# An American Text-Book of Genito-Urinary Diseases, Syphilis, and Diseases of the Skin

**Published:** 1899-03

**Authors:** 


**An American Text-Book of Genito-Urinary Diseases, Syphilis, and Diseases of the Skin.** Edited by L. Bolton Bangs, M.D., and W. A. Hardaway, M.D. Two volumes. Pp. 1—592, 593—1229. London: The Rebman Publishing Co. (Ltd.). Philadelphia: W. B. Saunders. 1898.

The combination of genito-urinary diseases with dermatology, or even with syphilis, is rather astonishing to English readers, but seems to be quite a usual trans-Atlantic practice, as there is an American *Journal of Cutaneous and Genito-Urinary*

*Diseases*. The articles in this work are by various writers, some of whom are well known for their contributions to the literature of the subject on which they write. Dr. White, of Philadelphia, for instance, writes on diseases of the prostate in conjunction with Dr. Wood, and his recent remarks on the treatment of prostatic hypertrophy by castration and vasectomy are of particular value. It is curious to find "inflammation of the seminal vesicle, the cord, and epididymis, and the testicle proper" as the heading of a section of a chapter, but the author evidently attaches great importance to diseases of the seminal vesicles, and their diagnosis. The subject of diseases and injuries of the ureter is, as might be expected in a modern work, treated in a separate chapter, and by so able a writer as Christian Fenger.

The more recent views about ringworm—including Sabouraud's—are clearly put forth; but ultimately, we feel sure, they will be modified: and the treatment of that comprehensive disease, eczema, is good and rings with common sense. We read: "The successful treatment of eczema is something more than the hasty prescription . . . for internal use and a salve or lotion for external application," and we note with strong approval that all eczemas should be cured. "Internal treatment . . . is not called for in a large proportion of the cases," and "it may be expressed as almost a law that the more circumscribed an eczematous eruption is the less will be needed in the form of general medication; . . . local eczemas require local applications." We have still the long-standing dispute about lichen ruber acuminatus, lichen ruber, and lichen ruber planus, and it "bids fair to afford abundant material for discussion for some time to come."

The work contains a very thorough and valuable collection of information on the subject of genito-urinary surgery, and the numerous illustrations are good, in this portion particularly those of hypertrophy of the prostate gland, in connection with the article by Drs. White and Wood, while the coloured plates and other illustrations of skin diseases are very faithfully and artistically executed; some of them are the best we have seen in any similar kind of work. We commend the book very heartily. There are many very useful prescriptions and the classification of skin affections is as good as it can be with the present nomenclature.

